# Shifting from glucose diagnosis to the new HbA1c diagnosis reduces the capability of the Finnish Diabetes Risk Score (FINDRISC) to screen for glucose abnormalities within a real-life primary healthcare preventive strategy

**DOI:** 10.1186/1741-7015-11-45

**Published:** 2013-02-21

**Authors:** Bernardo Costa, Francisco Barrio, Josep L Piñol, Joan J Cabré, Xavier Mundet, Ramon Sagarra, Jordi Salas-Salvadó, Oriol Solà-Morales

**Affiliations:** 1 Jordi Gol Primary Care Research Institute, Reus-Tarragona Diabetes Research Group, Catalan Health Institute, Primary Health Care Division, Camí de Riudoms 53-55, 43202, Reus, Spain; 2 Human Nutrition Unit, Faculty of Medicine and Health Sciences, Pere Virgili Health Research Institute. Rovira i Virgili University, Sant Llorenç 21, 43201, Reus, Spain; 3 CIBERobn Physiopathology of Obesity and Nutrition, Institute of Health Carlos III, Monforte de Lemos 5, 28029, Madrid, Spain; 4 Pere Virgili Health Research Institute. Health Institute Technology Transfer, Tarragona-Barcelona, Sant Llorenç 21, 43201, Reus, Spain

**Keywords:** Type 2 diabetes, screening, impaired fasting glucose, impaired glucose tolerance, pre-diabetes, FINDRISC, primary healthcare

## Abstract

**Background:**

To investigate differences in the performance of the Finnish Diabetes Risk Score (FINDRISC) as a screening tool for glucose abnormalities after shifting from glucose-based diagnostic criteria to the proposed new hemoglobin (Hb)A1c-based criteria.

**Methods:**

A cross-sectional primary-care study was conducted as the first part of an active real-life lifestyle intervention to prevent type 2 diabetes within a high-risk Spanish Mediterranean population. Individuals without diabetes aged 45-75 years (n = 3,120) were screened using the FINDRISC. Where feasible, a subsequent 2-hour oral glucose tolerance test and HbA1c test were also carried out (n = 1,712). The performance of the risk score was calculated by applying the area under the curve (AUC) for the receiver operating characteristic, using three sets of criteria (2-hour glucose, fasting glucose, HbA1c) and three diagnostic categories (normal, pre-diabetes, diabetes).

**Results:**

Defining diabetes by a single HbA1c measurement resulted in a significantly lower diabetes prevalence (3.6%) compared with diabetes defined by 2-hour plasma glucose (9.2%), but was not significantly lower than that obtained using fasting plasma glucose (3.1%). The FINDRISC at a cut-off of 14 had a reasonably high ability to predict diabetes using the diagnostic criteria of 2-hour or fasting glucose (AUC = 0.71) or all glucose abnormalities (AUC = 0.67 and 0.69, respectively). When HbA1c was used as the primary diagnostic criterion, the AUC for diabetes detection dropped to 0.67 (5.6% reduction in comparison with either 2-hour or fasting glucose) and fell to 0.55 for detection of all glucose abnormalities (17.9% and 20.3% reduction, respectively), with a relevant decrease in sensitivity of the risk score.

**Conclusions:**

A shift from glucose-based diagnosis to HbA1c-based diagnosis substantially reduces the ability of the FINDRISC to screen for glucose abnormalities when applied in this real-life primary-care preventive strategy.

## Background

Type 2 diabetes is a significant preventable disease and a growing public-health problem. When planning diabetes-prevention measures, people at risk for the disease should be targeted with lifestyle-modification interventions through a stepwise high-risk approach tailored to the specific local situation [1]. Simple prediction tools that can identify at-risk individuals could reduce the cost and inconvenience of screening. With such tools, a two-step procedure could be used: first, patients would be screened with a risk score; and second, those individuals identified to have a high risk for diabetes, would have their glycemic status assessed by measuring fasting plasma glucose (FPG), either alone or along with 2-hour post-load glucose (2hPG) using the oral glucose tolerance test (OGTT), or the more recently authorized hemoglobin (Hb)A1c measurement [2-4].

The notion that diabetes development can be prevented or delayed by intensive lifestyle intervention is not new [5,6]. However, it has recently been suggested that progression to diabetes can be also delayed by intensive intervention when applied to real-life primary health care of high-risk subjects identified first with the simple Finnish Diabetes Risk Score (FINDRISC) tool [7]. If such a risk score can be shown to have general applicability, it could provide a rational basis to decide which patients might benefit from intensive lifestyle intervention [8]. Thus, community-based evaluations are essential in order to learn about the FINDRISC feasibility and performance to screen for current and future glucose disorders.

The present study aimed at assessing possible differences in the performance of the FINDRISC as a screening tool for glucose abnormalities after shifting from the previously agreed 2hPG and FPG diagnostic criteria to the new HbA1c criteria in a real-life primary healthcare strategy to prevent type 2 diabetes within a Spanish Mediterranean population.

## Methods

### Ethics approval

The research ethics committee board at the Jordi Gol Research Institute (Barcelona, Spain) approved the protocol, and all participants gave written informed consent.

### Training, data sources, and study participants

The methods described for the active public-health program, DE-PLAN (Diabetes in Europe-Prevention using Lifestyle, Physical Activity and Nutritional intervention), which was developed in Catalonia (DE-PLAN-CAT), were used for this study [9]. All participating professionals were certified before recruitment, after attending several training meetings.

White Europeans without diabetes aged 45-75 years were evaluated by general practitioners in 18 primary healthcare centers. These participating centers were selected in a stratified manner, and covered all primary-care services for 315,703 inhabitants (4.5% of the population in Catalonia). The participants were consecutively recruited from a random list of the computerized public-healthcare system to obtain a representative sample of the population assigned to each center. For the associated lifestyle intervention study (at least 5-year follow-up of the screened subjects) all individuals with conditions such as severe psychiatric disease (for example, such as bipolar disorder or psychosis), chronic kidney disease (severe chronic renal failure) and serious chronic liver disease, or blood disorders (for example, severe iron-deficiency anemia or other conditions that might interfere with the HbA1c measurement), were excluded from the study.

The first screening used the Spanish version of the FINDRISC, a well-validated, eight-item European questionnaire related to diabetes risk factors to characterize subjects according to their future risk of type 2 diabetes. The most recent version was used, and ranged from 0 to 26 points, as follows: <7 points (low), 7 to 11 (slightly increased), 12 to 14 (moderate), 15 to 20 (high), and over 20 (very high) [10]. Both the questionnaire and the European guidelines recommend that scores of 15 or higher should prompt blood tests for diabetes [9-12]. The questionnaire collected information about age, sex, weight and height (to calculate body mass index; BMI), waist circumference, use of concomitant blood-pressure medication, history of high blood glucose disorders, physical activity, family history of diabetes, and daily consumption of vegetables, fruits and berries. Body weight and height were measured in light clothing, without shoes. Waist circumference was measured midway between the lowest rib and the iliac crest. Anthropometric parameters were determined by trained nurses.

The second screen involved use of a 2-hour 75-g OGTT, in accordance with the World Health Organization (WHO) standards, along with measurements of FPG and 2hPG, carried out in all recruiting centers. All participants with FINDRISC scores of 15 or over were asked to undergo a screening OGTT as part of the protocol. Participants with FINDRISC scores below 15 were also offered an OGTT if they wished [7]. For this part of the DE-PLAN-CAT project (screening), diagnosis of all glucose disorders was based on the results of a single OGTT. Any volunteer with either a FPG or 2hPG result suggestive of diabetes was excluded from participation in the subsequent part of the project (lifestyle intervention). A second OGTT to confirm the diagnosis of diabetes was recommended in the study protocol for those individuals who did ultimately participate in the lifestyle intervention.

Plasma glucose level was determined by a uniform glucose oxidase-peroxidase method. HbA1c determination was performed at the same time, using a standardized high-performance liquid chromatography (HPLC) assay aligned to the Diabetes Control and Complications Trial in all laboratories [13]. Blood samples were analyzed using similar techniques at five laboratories, four of which were based in the same institution (Catalan Health Institute). The intra-assay and interassay coefficients of variation for all assays ranged from 2 to 3%.

Three main diagnostic categories (normal, pre-diabetes and diabetes) were defined using the WHO criteria based on 2hPG (less than 7.8, 7.8 to 11.0 mmol/l and greater or equal than 11.1 mmol/l) and/or FPG (6.1 to 6.9 mmol/l); the American Diabetes Association (ADA) criteria based on FPG (less than 5.5, 5.5 to 6.9, and greater or equal than 7.0 mmol/l); and the new proposed HbA1c criteria (less than 38, 38 to 48, and greater than 48 mmol⁄mol) or (less than 5.7, 5.7 to 6.4, and greater than 6.4%). The diagnostic categories derived from these alternative approaches were compared with the FINDRISC test scores and risk classes in order to investigate the capability of the questionnaire in classifying individuals according to their current glycemic status.

### Statistical analysis

The sample size calculation details using available data on diabetes incidence in the high-risk Catalan population have been published previously [7]. Assuming that the population to be screened were able to accept the proposal to participate in the subsequent lifestyle intervention phase, we calculated that the study needed at least 1,650 people in the screening period (type 1 and type 2 errors: 5% and 20%, respectively). Multiple comparisons of the significant differences between groups were carried out by one-way ANOVA, and/or by Student's *t*-test for continuous variables and the χ^2 ^test for categorical variables. The main results are presented using the WHO criteria as the current standard, compared with the ADA criteria and the new HbA1c criteria.

Given the stratification of the sample, a pooled analysis of all questionnaires was conducted. Sensitivity, specificity, and predictive values were calculated for different cut-off points of the FINDRISC test and for different sets of diagnostic criteria. The positive and the negative predictive values (PPV and NPV) and respective likelihood ratios (LRs) were also calculated. The 95% confidence interval (CI) for sensitivity, specificity, predictive values, and LRs was estimated. To determine the performance of the questionnaire and the optimal FINDRISC cut-off point for the detection of diabetes and all glucose abnormalities (diabetes and pre-diabetes), the receiver operating characteristic (ROC) curves were calculated by plotting the sensitivity of the test versus the false-positive rate (1 minus specificity). The optimal cut-off points used were the peaks of the curve, where the sum of sensitivity and specificity is at maximum. The area under the ROC curve (AUC) with its 95% CI was used to compare results between the three sets of diagnostic criteria based on 2hPG, FPG and HbA1c, respectively. Statistical analyses were conducted using SPSS for Windows (version 15.0; SPSS Inc., Chicago, IL, USA).

## Results

In all, 3,647 subjects were invited to participate (79% by direct contact and 21% by phone) of whom 3,120 (85.5%) accepted the invitation to the first screening session, which used the FINDRISC. In this group, 65.5% were women, the mean age was 60.1 years, and the mean BMI was 28.8 kg/m^2^. Most of the FINDRISC questionnaires were filled in by the healthcare providers during the first interview (95%), but a small number were self-administered (5%). The main characteristics of the participants in this first step including their FINDRISC findings are reported in Table 1. Given the usually recommended cut-off point of 15, the questionnaire identified 40 individuals (26.9%) as having high or very high risk of diabetes.

**Table 1:** Characteristics of the participants in the first screening step (n = 3,120) by sex including Finnish Diabetes Risk Score (FINDRISC) findings

Parameter	Overall	Male	Female	*p*
Number of participants, n	3120	1077 (34.5)	2043 (65.5)	-
Age, years	60.1 ± 8.3	61.3 ± 8.3	59.5 ± 8.2	<0.01
45 to 54	941 (30.2)	275 (25.5)	666 (32.6)	
55 to 64	1157 (37.1)	377 (35.0)	780 (38.2)	
>65	1022 (32.8)	425 (39.5)	597 (29.2)	
BMI, kg/m^2^	28.8 ± 4.6	28.7 ± 4.0	28.9 ± 4.9	0.38
<25	612 (19.6)	185 (17.2)	427 (20.9)	
25 to 30	1464 (46.9)	555 (51.5)	909 (44.5)	
≥30	1044 (33.5)	337 (31.3)	707 (34.6)	
Waist circumference, cm	95.6 ± 11.5	100.0 ± 10.0	93.4 ± 11.6	<0.01
M <94, F <80	517 (16.6)	256 (23.8)	261 (12.8)	
M 94 to 102, F 80 to 88	942 (30.2)	402 (37.7)	540 (26.4)	
M ≥103, F ≥89	1661 (53.2)	419 (38.9)	1242 (60.8)	
Systolic blood pressure, mmHg	131.7 ± 15.4	134.0 ± 14.7	130.5 ± 15.7	<0.01
Diastolic blood pressure, mmHg	78.2 ± 10.6	79.0 ± 9.3	77.8 ± 11.1	<0.01
FINDRISC, points	11.8 ± 4.5	11.4 ± 4.4	12.0 ± 4.6	<0.01
Low risk (<7)	346 (11.1)	136 (12.6)	210 (10.3)	
Slightly increased risk (7 to 11)	1221 (39.1)	441 (40.9)	780 (38.2)	
Moderate risk (12 to 14)	713 (22.9)	235 (21.8)	478 (23.4)	
High risk (15 to 20)	741 (23.8)	244 (22.7)	497 (24.3)	
Very high risk (>20)	99 (3.2)	21 (1.9)	78 (3.8)	

^a^Data are means ± SE for quantitative variables or n (%) for qualitative variables.

Of the original 3,120 participants in the first screen, 1,746 participants (56%) also authorized the second screening session, using blood tests; of these, 1,712 (54.9%) cases had all requested data available. In this group, 66.8% were women, the mean age was 60.7 years, and the mean BMI was 29.7 kg/m^2^. The characteristics of the participants in this second step, including glucose and HbA1c diagnostic findings, are reported in Table 2. In all, 723 individuals (42.2%) who underwent blood testing had been previously classified by the questionnaire as having high or very high risk of diabetes. We found no significant difference in the FINDRISC items between subjects who accepted or rejected the blood test. The risk of diabetes assessed by the FINDRISC score was higher in women, whereas the risk of diabetes assessed by either the glucose or the HbA1c measurements was higher in men.

**Table 2:** Characteristics of the participants in the second screening step (n = 1,712) by sex including Finnish Diabetes Risk Score (FINDRISC), glucose and hemoglobin (Hb)A1c findings

Parameter	Overall	Male	Female	*p*
Number of participants (n)	1712	569 (33.2)	1143 (66.8)	-
Age, years	60.7 ± 8.2	62.4 ± 8.1	59.8 ± 8.1	<0.01
45 to 54	464 (27.1)	124 (21.8)	340 (29.7)	
55 to 64	640 (37.4)	187 (32.9)	453 (39.6)	
>65	608 (35.5)	258 (45.3)	350 (30.6)	
BMI, kg/m^2^	29.7 ± 4.7	29.5 ± 3.9	29.7 ± 5.1	0.43
<25	240 (14.0)	59 (10.4)	181 (15.8)	
25 to 30	740 (43.2)	274 (48.2)	466 (40.8)	
≥30	732 (42.8)	236 (41.5)	496 (43.4)	
Waist circumference, cm	97.4 ± 11.5	102.0 ± 9.8	95.2 ± 11.6	<0.01
M <94, F <80	193 (11.3)	87 (15.3)	106 (9.3)	
M 94 to 102, F 80 to 88	463 (27.0)	205 (36.0)	258 (22.6)	
M ≥103, F ≥89	1056 (61.7)	277 (48.7)	779 (68.2)	
Systolic blood pressure, mmHg	132.6 ± 15.5	135.0 ± 14.8	131.5 ± 15.7	<0.01
Diastolic blood pressure, mmHg	78.8 ± 11.4	79.2 ± 9.4	78.6 ± 12.4	0.33
FINDRISC, items	13.5 ± 4.4	13.2 ± 4.2	13.7 ± 4.5	0.04
Low risk (<7)	98 (5.7)	29 (5.1)	69 (6.0)	
Slightly increased risk (7 to 11)	475 (27.7)	174 (30.6)	301 (26.3)	
Moderate risk (12 to 14)	416 (24.3)	135 (23.7)	281 (24.6)	
High risk (15 to 20)	633 (37.0)	212 (37.3)	421 (36.8)	
Very high risk (>20)	90 (5.3)	19 (3.3)	71 (6.2)	
2-hour Plasma glucose, mmol/l	6.8 ± 2.8	7.4 ± 3.0	6.6 ± 2.7	<0.01
Diabetes	158 (9.2)	70 (12.3)	88 (7.7)	
Pre-diabetes	380 (22.2)	167 (29.3)	213 (18.6)	
Fasting plasma glucose, mmol/l	5.2 ± 1.0	5.4 ± 1.1	5.1 ± 1.0	<0.01
Diabetes	53 (3.1)	22 (3.9)	31 (2.7)	
Pre-diabetes	432 (25.2)	189 (33.2)	243 (21.3)	
HbA1c (NGSP),%^b^	5.5 ± 0.6	5.5 ± 0.6	5.5 ± 0.6	0.26
HbA1c (IFCC), mmol/mol	36.3 ± 6.4	36.6 ± 6.7	36.2 ± 6.3	0.26
Diabetes	62 (3.6)	23 (4.0)	39 (3.4)	
Pre-diabetes	429 (25.1)	149 (26.2)	280 (24.5)	

^a^Data are means ± SE for quantitative variables or n (%) for qualitative variables.

^b^HbA1c values have been indicated in accordance with both the National Glycohemoglobin Standardization Program (NGSP) and the International Federation of Clinical Chemistry and Laboratory Medicine (IFCC) criteria.

**Figure 1:**
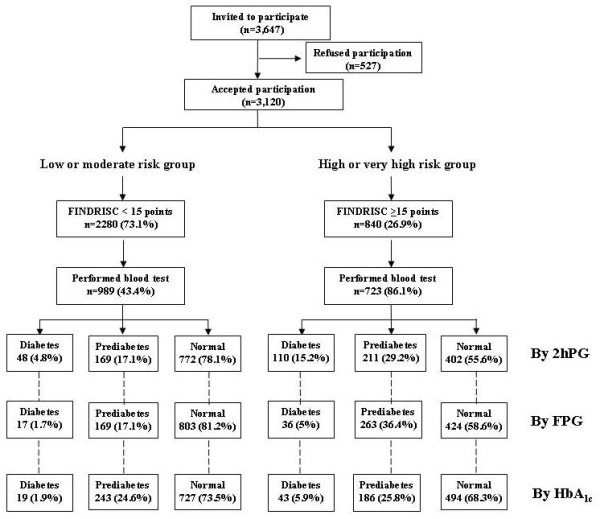
**Distribution of participation in the screening**. Flowchart of the two screening phases, showing the distribution of participants by Finnish Diabetes Risk Score (FINDRISC) results and the three sets of diagnostic criteria: 2-hour plasma glucose (2hPG), fasting plasma glucose (FPG), and hemoglobin (Hb)A1c.

The diagnostic categories by the WHO criteria (which includes 2hPG) were 1,174 (68.6%; 95% CI 66.3 to 70.8) individuals with normal glucose tolerance, 380 (22.2%; 95% CI 20.2 to 24.2) with pre-diabetes, and 158 (9.2%; 95% CI 7.9 to 10.7) with diabetes. The corresponding FPG-based findings (ADA criteria) were 1,227 (71.7%; 95% CI 69.5 to 73.8) with normal fasting glucose, 432 (25.2%; 95% CI 23.2 to 27.4) with pre-diabetes, and 53 (3.1%; 95% CI 2.3 to 4.0) with diabetes. Findings based on HbA1c were 1,221 (71.3%; 95% CI 69.1 to 73.4) with normal HbA1c, 429 (25.1%; 95% CI 23.0 to 27.2) with pre-diabetes, and 62 (3.6%; 95% CI 2.8 to 4.6) with diabetes. Figure 1 summarizes both of the screening phases and the participant distribution by test results and diagnostic criteria. Of the subgroup of subjects identified as being high or very high risk (n = 723) by the FINDRISC, the 2hPG, FPG, and HbA1c tests indicated 29.2%, 36.4%, and 25.8%, respectively as having pre-diabetes and 15.2%, 5.0%, and 5.9% as having diabetes. Meanwhile, these findings were 17.1%, 17.1%, and 24.6%, respectively, for pre-diabetes and 4.8%, 1.7%, and 1.9% for diabetes in the individuals identified by the FINDRISC as having low, slightly increased, or moderate risk (n = 989).

**Figure 2:**
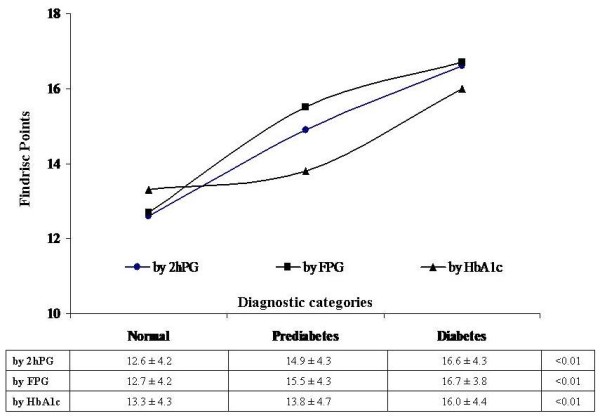
**Distribution of test scores by diagnostic categories**. Finnish Diabetes Risk Score (FINDRISC) values classified by glucose-metabolism category (normal, pre-diabetes, diabetes), using the 2-hour plasma glucose (2hPG), fasting plasma glucose (FPG) and hemoglobin (Hb)A1c diagnostic criteria. Data in the associated table are mean ± SD.

Mean FINDRISC values showed a progressive and significant increase (*P*<0.01) as the glucose-metabolism categories worsened (normal, pre-diabetes, diabetes), regardless of the set of diagnostic criteria applied, whether 2hPG, FPG, or HbA1c (Table 3). Thus, we found a strong correlation between FINDRISC classes and either the glucose or the HbA1c values, and this tended to increase dramatically with increasing FINDRISC scores (Figure 2). Likewise, individuals with higher scores had higher values for age, BMI, waist circumference, and systolic and diastolic blood pressure (Table 3). No significant differences between the recruiting centers regarding the FINDRISC test scores or the distribution of the risk classes were found.

**Table 3:** Characteristics of the participants in the second screening step (n = 1,712) by Finnish Diabetes Risk Score (FINDRISC) classes including glucose and hemoglobin (Hb)A1c findings

Parameter	FINDRISC classes					Total	*p*
			
	<7	7 to 11	12 to 14	15 to 20	>20		
Number of participants, n	98 (5.7)	475 (27.7)	416 (24.3)	633 (37.0)	90 (5.3)	1712	-
Sex, men	29 (29.6)	174 (36.6)	135 (32.5)	212 (33.5)	19 (21.1)	569 (33.2)	0.06
Age, years	55.0 ± 7.5	60.1 ± 8.2	60.4 ± 8.0	62.0 ± 8.0	61.8 ± 8.4	60.7 ± 8.2	<0.01
45-54	53 (54.1)	147 (30.9)	114 (27.4)	129 (20.4)	21 (23.3)	464 (27.1)	
55-64	34 (34.7)	172 (36.2)	160 (38.5)	244 (38.5)	30 (33.3)	640 (37.4)	
>65	11 (11.2)	156 (32.8)	142 (34.1)	260 (41.1)	39 (43.3)	608 (35.5)	
BMI, kg/m^2^	23.8 ± 2.4	27.6 ± 3.9	29.7 ± 4.5	31.5 ± 4.2	33.6 ± 4.4	29.7 ± 4.7	<0.01
< 25	70 (71.4)	107 (22.5)	41 (9.9)	19 (3.0)	3 (3.3)	240 (14.0)	
25-30	27 (27.6)	281 (59.2)	208 (50.0)	215 (34.0)	9 (10.0)	740 (43.2)	
≥ 30	1 (1.0)	87 (18.3)	167 (40.1)	399 (62.7)	78 (86.7)	732 (42.8)	
Waist circumference, cm	81.7 ± 7.7	93.2 ± 10.6	97.9 ± 10.8	101.8 ± 9.8	105.2 ± 9.6	97.4 ± 11.5	<0.01
M<94, F<80	72 (73.5)	86 (18.1)	24 (5.8)	11 (1.7)	0 (0.0)	193 (11.3)	
M 94-102, F 80-88	26 (26.5)	186 (39.2)	113 (27.2)	137 (21.6)	1 (1.1)	463 (27.0)	
M ≥103, F ≥89	0 (0.0)	203 (42.7)	279 (67.1)	485 (76.6)	89 (98.9)	1056 (61.7)	
Systolic blood pressure, mmHg	122.8 ± 14.5	130.7 ± 15.1	132.7 ± 15.7	134.8 ± 14.9	138.3 ± 16.7	132.6 ± 15.5	<0.01
Diastolic blood pressure, mmHg	73.7 ± 7.9	77.4 ± 8.7	79.4 ± 16.8	79.7 ± 9.1	81.9 ± 9.2	78.8 ± 11.4	<0.01
FINDRISC items							
Antihypertensive medication	6 (6.1)	136 (28.6)	170 (40.9)	394 (62.2)	66 (73.3)	772 (45.1)	<0.01
Physical activity	81 (82.7)	389 (81.9)	296 (71.2)	416 (65.7)	36 (40.0)	1218 (71.1)	<0.01
Consumption of vegetables/fruits	81 (82.7)	428 (90.1)	358 (86.1)	540 (85.3)	75 (83.3)	1482 (86.6)	0.09
History of blood glucose disorders	0 (0.0)	17 (3.6)	60 (14.4)	277 (43.8)	88 (97.8)	442 (25.8)	<0.01
Family history, first degree	4 (4.1)	46 (9.7)	65 (15.6)	74 (11.7)	4 (4.4)	193 (11.3)	<0.01
Family history, other degree	0 (0.0)	88 (18.5)	172 (41.3)	404 (63.8)	83 (92.2)	747 (43.6)	<0.01
2-hour Plasma glucose, mmol/l	5.3 ± 2.0	6.2 ± 2.3	6.4 ± 2.6	7.4 ± 3.0	9.4 ± 3.2	6.8 ± 2.8	<0.01
Diabetes	1 (1.0)	24 (5.1)	23 (5.5)	81 (12.8)	29 (32.2)	158 (9.2)	
Pre-diabetes	7 (7.1)	87 (18.3)	75 (18.0)	172 (27.2)	39 (43.3)	380 (22.2)	
Fasting plasma glucose, mmol/l	4.7 ± 0.5	5.0 ± 0.8	5.1 ± 1.0	5.4 ± 1.1	6.0 ± 1.3	5.2 ± 1.0	<0.01
Diabetes	0 (0.0)	7 (1.5)	10 (2.4)	28 (4.4)	8 (8.9)	53 (3.1)	
Pre-diabetes	6 (6.1)	83 (17.5)	80 (19.2)	210 (33.2)	53 (58.9)	432 (25.2)	
HbA1c (NGSP),%^b^	5.3 ± 0.5	5.4 ± 0.5	5.4 ± 0.6	5.5 ± 0.6	5.8 ± 0.5	5.5 ± 0.6	<0.01
HbA1c (IFCC), mmol/mol^b^	34.3 ± 5.3	35.2 ± 5.9	36.0 ± 6.2	37.1 ± 6.9	40.1 ± 5.8	36.3 ± 6.4	<0.01
Diabetes	1 (1.0)	9 (1.9)	9 (2.2)	33 (5.2)	10 (11.1)	62 (3.6)	
Pre-diabetes	22 (22.4)	114 (24.0)	107 (25.7)	150 (23.7)	36 (40.0)	429 (25.1)	

^a^Data are means ± SE for quantitative variables or n (%) for qualitative variables.

^b^HbA1c values have been indicated in accordance with both the National Glycohemoglobin Standardization Program (NGSP) and the International Federation of Clinical Chemistry and Laboratory Medicine (IFCC) criteria.

**Figure 3:**
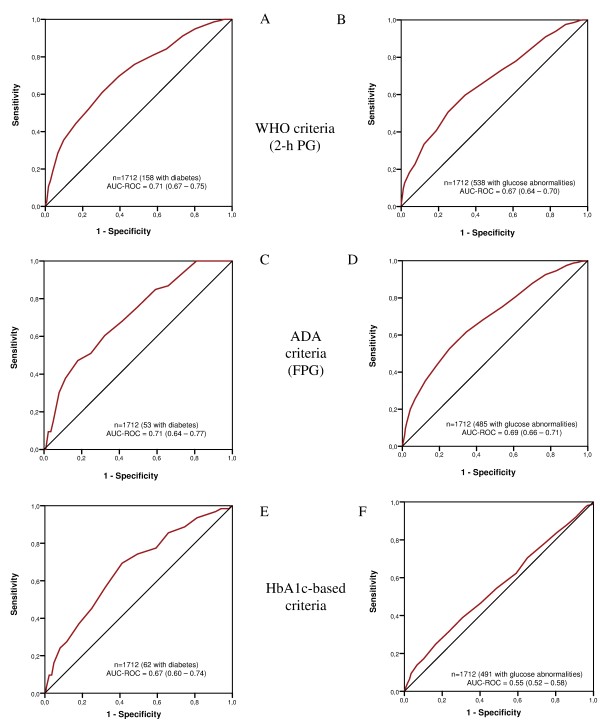
**Receiver operating characteristic (ROC) curves by glucose and hemoglobin (Hb)A1c diagnosis**. Receiver operating characteristic curves for the prevalence of **(A, C, E) **unknown type 2 diabetes and **(B, D, F) **overall glucose abnormalities (diabetes and pre-diabetes) classified by the 2-hour plasma glucose (2hPG), fasting plasma glucose (FPG) and hemoglobin (Hb)A1c diagnostic criteria.

The ROC curves for detecting unknown diabetes and any other degree of abnormal glucose metabolism (diabetes and pre-diabetes) in the studied sample by the diagnostic criteria applied were calculated (Figure 3). Table 4 shows the FINDRISC findings (sensitivity, specificity, and predictive values) using different cut-off points for screen-detected diabetes and overall glucose metabolic abnormalities, allowing for all diagnostic criteria. The ROC curves indicated that a cut-off of 14 for detecting glucose-metabolism abnormalities offered the best balance between true-positive and false-positive rates in this population, irrespective of the set of diagnostic criteria used.

**Table 4:** Characteristics of the main Finnish Diabetes Risk Score (FINDRISC) cut-off points for screening-detected type 2 diabetes and glucose abnormalities (diabetes and pre-diabetes), classified by glucose and hemoglobin (Hb)A1c diagnostic criteria.

Cut-off values	n (%)	Diabetes^a^				Glucose abnormalities^a^			
		
		Sensitivity	Specificity	PPV	NPV	Sensitivity	Specificity	PPV	NPV
Diagnostic classification by the WHO criteria (2-hour plasma glucose)									
6	31 (1.8)	100.0	4.3	9.6	100.0	99.3	5.4	32.5	94.0
10	111 (6.5)	94.9	19.7	11.7	97.5	90.9	22.6	35.0	84.4
13	166 (9.7)	81.0	42.1	12.5	95.6	73.2	46.0	38.3	78.9
14	139 (8.1)	75.9	52.3	13.9	95.5	65.8	56.7	41.1	78.4
15	156 (9.1)	69.6	60.6	15.2	95.1	59.7	65.8	44.4	78.1

Diagnostic classification by the ADA criteria (fasting plasma glucose)									
6	31 (1.8)	100.0	4.0	3.2	100.0	99.2	5.1	29.2	94.0
10	111 (6.5)	100.0	18.9	3.8	100.0	92.6	22.7	32.1	88.5
13	166 (9.7)	84.9	40.7	4.4	98.8	75.3	46.0	35.5	82.5
14	139 (8.1)	75.5	50.5	4.6	98.5	68.0	56.6	38.3	81.8
15	156 (9.1)	67.9	58.6	5.0	98.3	61.6	65.4	41.4	81.2

Diagnostic classification by the HbA1c-based criteria									
6	31 (1.8)	98.4	4.0	3.7	98.5	97.1	4.3	29.0	79.1
10	111 (6.5)	93.5	18.8	4.2	98.7	83.9	19.2	29.5	74.8
13	166 (9.7)	77.4	40.6	4.7	98.0	62.3	40.9	29.8	73.0
14	139 (8.1)	74.2	50.5	5.3	98.1	54.4	51.3	31.0	73.6
15	156 (9.1)	69.4	58.8	5.9	98.1	46.6	59.5	31.7	73.5

ADA, American Diabetic Association; Hb, hemoglobin; NPV, negative predictive value; PPV, positive predictive value.

^a^Data on sensitivity, specificity, PPV and NPV are percentages.

Drawing on this cut-off point of greater than or equal to 14 on the FINDRISC scale, and regarding the diagnostic classification by the WHO criteria (which includes 2hPG), we found that the AUC for detecting unknown diabetes (Figure 3A) was 0.67 (95% CI 0.59 to 0.72) for men and 0.76 (95% CI 0.70 to 0.81) for women. The corresponding values for all glucose abnormalities (Figure 3B) were 0.64 (95% CI 0.60 to 0.69) for men and 0.70 (95% CI 0.66 to 0.73) for women. The sensitivity and specificity were 75.9% and 52.3% for the detection of type 2 diabetes alone, and 65.8% and 56.7% for detecting any degree of abnormal glucose metabolism. The cut-off point of 14 had an NPV of 95.5% for diabetes and 78.4% for glucose abnormalities (Table 4).

For FPG, the corresponding values (ADA criteria) for diabetes (Figure 3C) were 0.72 (95% CI 0.63 to 0.82) for men and 0.70 (95% CI 0.61 to 0.79) for women. Equivalent values for all glucose metabolic abnormalities (Figure 3D) were 0.64 (95% CI 0.60 to 0.69) and 0.73 (95% CI 0.69 to 0.76), respectively. For a FINDRISC greater or equal to 14, the sensitivity and specificity were 75.5% and 50.5%, respectively, for the detection of diabetes, and 68.0% and 56.6%, respectively, for detecting glucose abnormalities. The NPVs were 98.5% and 81.8%, respectively (Table 4).

Finally, parallel findings based on HbA1c for diabetes (Figure 3E) were 0.62 (95% CI 0.53 to 0.70) for men and 0.70 (95% CI 0.61 to 0.80)] for women. The corresponding values for all glucose metabolic abnormalities (Figure 3F) were 0.51 (95% CI 0.46 to 0.57) and 0.57 (95% CI 0.53 to 0.61), respectively. For a cut-off point of 14, the sensitivity and specificity were 74.2% and 50.5%, respectively, for the detection of diabetes, and 54.4% and 51.3% respectively, for detecting glucose abnormalities. The corresponding NPVs were 98.1% and 73.6%, respectively (Table 4).

## Discussion

### Screening for diabetes and prevention programs

The growing prevalence of type 2 diabetes requires the development and introduction of better prevention strategies to reduce the incidence and prevalence of the disease [14]. Regrettably, diabetes prevention has not been prioritized worldwide, despite clear evidence that not including these policies results not only in health costs, but also other costs to society. Although the development of specific preventive measures for diabetes that target the entire population is not an appropriate strategy, it is essential to identify subjects at increased risk; consequently, a simple, inexpensive, non-invasive and valid tool focused on classic and valuable risk factors is needed [8].

It is currently recommended that screening for diabetes and pre-diabetes should be carried out using a risk score, followed by conventional diagnosis in those individuals identified as being at high risk. HbA1c is a good marker of protein glycation secondary to long-term exposure to glucose, but until recently, it had not been considered for this purpose [15]. While the 2-hour, fasting glucose, and HbA1c can all be defined as continuous statistical variables that are influenced by individual habits, it is not well known which of these variables are best related to the primary risk factors of diabetes [16]. At present, the FINDRISC, which is the most accurate and widely questionnaire used in Europe, can easily identify people with either unrecognized diabetes or impaired glucose regulation, before any blood test needs to be carried out [9].

During the past decade, many studies whose common purpose was the validation of different questionnaires to predict current or future diabetes have been published. In all these studies, diagnoses were assessed using the glucose-based criteria [17-19]. Although the majority of these studies included non-invasive variables (modifiable or not) that can be easily obtained (obesity, aging, family and personal history), other studies used biochemical (blood glucose, lipid profile, insulinemia, biomarkers) or even genetic variables (polymorphisms) in an attempt to increase their performance. Clearly, from the standpoint of primary health are, those using non-invasive variables are the most suitable because they simplify the task of screening in daily clinical practice. We consider that the target population for community prevention programs should not be limited only to individuals with impaired glucose tolerance, despite the strong scientific evidence for the effectiveness of preventive measures in individuals with this diagnosis. Much work has been carried out to develop diabetes risk scores, but most are rarely used because they require blood tests that are not routinely available. Furthermore, it has been shown that using more complex variables adds little or nothing to the overall model, and does not always improve the performance of the risk score [20,21].

### Issues and limitations in screening for diabetes by the FINDRISC in primary care

The DE-PLAN-CAT cohort was prospectively recruited for the express purpose of evaluating the FINDRISC questionnaire as an earlier detection tool for individuals at high risk for diabetes who would then be offered a preventive intervention. Obviously, a limitation of the present study is that it includes only data obtained during a large screen conducted in primary health care, not on prospective data based on future diagnoses, which will require a longer follow-up. In fact, the diagnosis of diabetes and pre-diabetes were based on only one OGTT value, not two, but this is a commonly accepted procedure for screening large samples. We tried first to measure the FINDRISC performance in predicting current glucose disorders and then to compare the results based on different sets of diagnostic criteria. Obviously, we cannot exclude the possibility of some selection bias. Nevertheless, the available data on the 4-year incidence of diabetes in the DE-PLAN-CAT cohort based on repeat testing have been contributed together with those from the derivation PREDIMED cohort to develop a new questionnaire tailored to the needs of our own Spanish Mediterranean setting [22].

The general profile of the participants was similar to that of the general populaiton attending primary care. As shown previously, women are most likely to use these services in Spain [7], and this predominance is similar to previous widespread trials concerning diabetes prevention in Finland and the USA [5,6]. Similar to these trials, the number of men in our trial was lower than the number of women, and the proportion of men aged over 65 years was greater than the proportion of included women of the same age. This distribution could perhaps explain why the risk of diabetes assessed by the score was higher in women whereas the risk assessed by the blood tests was higher in men.

In this regard, the age and sex distribution could be perceived as another bias at work, particularly if compared with larger population-based studies. Undoubtedly, the main reason for this is that the protocol was conducted under real working conditions in primary care; however this could also be considered as an advantageous approach for this study. Moreover, it seems that individuals identified as high risk at screening can all benefit similarly from lifestyle intervention, regardless of age, sex, and socioeconomic group [23]. In previous controlled trials, older people seemed to benefit somewhat more than younger ones, but men and women both had similar outcomes. Accordingly, in specifying the target participant profile for diabetes prevention in primary care, it does not seem necessary to pay too much attention to population subgroups; rather, it is more important to plan properly for consistent preventive measures [23-25].

### Limitations of the FINDRISC using HbA1c as diagnostic criterion

When estimating the overall discriminatory strength of the questionnaire by means of the AUCs, the FINDRISC produced values ranging from 0.72 to 0.86, at least in the Finnish derivation samples [8,23]. The performance of the Spanish version used in this study was 0.71 for detecting diabetes (both 2hPG and FPG diagnosis) and 0.67 (2hPG-based diagnosis) or 0.69 (FPG-based diagnosis) for detecting all glucose metabolic abnormalities (that is, diabetes and pre-diabetes). These figures are comparable with those obtained in most European countries other than Finland (validation samples), generally ranging from 0.60 to 0.80 [17-19]. Running the score on a new population with similar but not identical features from the population for which it was developed almost invariably leads to a loss of performance, suggesting that the FINDRISC questionnaire, although acceptable, should be validated within the population for which it is intended to be used.

However, almost all previous studies were carried out using the conventional diagnostic criteria based on glucose, and did not use the new diagnostic criteria based on HbA1c. When HbA1c was applied as the primary diagnostic criterion, the AUC dropped to 0.67 (5.6% reduction in comparison with either 2-hour or fasting glucose) for detecting diabetes, and in particular, it fell to 0.55 for detecting all glucose abnormalities (17.9% and 20.3% reduction, respectively). As far as we know, this is the first estimate of a possible loss of performance of the FINDRISC questionnaire if there is a widespread use of these new proposed HbA1c-based diagnostic criteria, at least as a screening tool in the context of a program aimed at preventing diabetes.

The ROC curves indicated that a lower cut-off of 14 for detecting diabetes or any glucose metabolic abnormality offered the best balance in this population, irrespective to the set of diagnostic criteria used. This cut-off is one point lower than 15, the most commonly used point [24], but even lower cut-off points have been considered suitable for screening in other community-based diabetes-prevention programs [25]. It is likely that if we had given blood tests to all participants who answered the FINDRISC questionnaire, the cut-off would also have increased. However, the strategy we used is a realistic one to identify individuals at high risk who might be offered a preventive intervention, rather than being a stringent experimental study aimed at validating the scale. In our study, we found that the maximum sensitivity and specificity of the FINDRISC were about 76% and 52%, respectively for detecting diabetes, and 68% and 56%, respectively, for detecting all glucose abnormalities. When diabetes was defined by a single HbA1c measurement, this resulted in a small decrease in sensitivity, ranging from 1.3% (compared with 2hPG-based diagnosis) to 1.7% (compared with FPG-based diagnosis). For detecting all glucose abnormalities, the use of HbA1c-based criteria led to a greater reduction in sensitivity, ranging from 11.4% (compared with FPG diagnosis) to 13.6% (compared with 2-hour plasma glucose diagnosis). The corresponding specificity findings also showed a reduction although this was more moderate, reaching a maximum of 5.4%.

In contrast to population-based studies, the DE-PLAN-CAT survey was essentially focused on a representative sample of undiagnosed subjects in primary care, where the likelihood of presenting glucose abnormalities obviously increases. Leaving aside the inconvenience of using the OGTT, both the WHO and the ADA criteria established that in the absence of unequivocal hyperglycemia, the results should be confirmed by repeat testing, at least in clinical practice. Therefore, it is not surprising that about one-third of the participants were diagnosed as having any type of impaired glucose metabolism, as the screening was based on a single test. In a previous work conducted in the same population, we showed that defining diabetes by FPG resulted in a significant decrease in prevalence compared with defining diabetes by 2hPG, even in repeat tests for those participants who agreed to the lifestyle intervention [26]. In addition, a shift from glucose-based to HbA1c-based diagnosis was also shown to reduce the apparent diabetes prevalence, with a low overall or single degree of overlap between diagnostic categories [26].

### Implications

The decrease in performance of the FINDRISC can be explained by the preceding validations using the glucose-based diagnostic criteria. A low level of diagnostic overlap and an underestimation of diabetes prevalence using the HbA1c mean that a number of individuals would be moved from the diabetes category to the normal or pre-diabetes categories. In fact, the proposed HbA1c cut-off point for diagnosing diabetes (48 mmol/mol or 6.5%) has resulted in minor discrepancies [27], but the cut-off point for pre-diabetes (38 mmol/mol or 5.7%) is under discussion, particularly with regard to its potential use in population screening [27]. Both the decrease in diabetes prevalence using the HbA1c and FINDRISC performance could have important implications for primary healthcare-based diabetes prevention.

## Conclusions

Our recommendation is that all people attending primary healthcare facilities should be screened for diabetes risk using the FINDRISC, perhaps with a lower cut-off point in mind, or preferably using a personal adapted score. The present study showed that the FINDRISC questionnaire, although a far from ideal tool, has a reasonably high capability of predicting current undiagnosed diabetes and pre-diabetes as defined by glucose-based diagnostic criteria in this cross-section of the Spanish population. However, a shift from the glucose-based diagnosis to the HbA1c-based diagnosis would significantly reduce not only the estimated diabetes prevalence but also the FINDRISC capability to screen for glucose abnormalities. Consequently, it is desirable that new adaptations of this score consider the real possibility of diagnosing by the HbA1c.

## Abbreviations

2hPG: 2-hour plasma glucose; AUC: area under the receiver operating characteristic curve; CI: confidence interval; FINDRISC: Finnish Diabetes Risk Score; FPG: fasting plasma glucose; HbA1c: Hemoglobin A1c; IFCC: International Federation of Clinical Chemistry; NGSP: National Glycohemoglobin Standardization Program; OGTT: oral glucose tolerance test; ROC: receiver operating characteristic.

## Competing interests

The authors declare that they have no competing interests.

## Authors' contributions

All authors substantially contributed to designing the study protocol or to data analysis and interpretation, and to drafting or revising the article. Specifically, BC wrote the national research proposal and the core of this manuscript; JLP and FB performed the statistical analysis; JJC and RS helped to draft the manuscript and particularly the subsequent revised versions; XM, JS, and OSM added significant contributions; and BC also organized the study within the structure of our public-health system. All authors read and approved the final version to be published.

## Pre-publication history

The pre-publication history for this paper can be accessed here:

http://www.biomedcentral.com/1741-7015/11/45/prepub
